# The Use of Orthostatic Device for 90 Minutes Does Not Change Cardiovascular and Biomechanical Parameters of Patients with Spinal Cord Injury

**DOI:** 10.1155/2022/3917566

**Published:** 2022-09-16

**Authors:** Liana Praça Oliveira, Reginaldo Florencio da Silva, Marie Aquino Melo de Leopoldino, Sarah Carvalho Frota, Gabriella Coelho Vieira de Melo Alves, Jefferson Pacheco Amaral Fortes, Pedro Henrique Gomes Muniz, Gisele Harumi Hotta, Francisco Carlos de Mattos Brito Oliveira, Francisco Fleury Uchoa Santos-Júnior

**Affiliations:** ^1^Physiotherapy Department, Centro Universitário Estácio do Ceará, Fortaleza, Brazil; ^2^Computation Sciences Department, State University of Ceará, Fortaleza, Brazil; ^3^Le Santé Institute, Fortaleza, Brazil; ^4^University of São Paulo, School of Medicine of Ribeirao Preto, Health Sciences Department, Ribeirão Preto, SP, Brazil

## Abstract

**Background:**

Changes in autonomic function are often caused by spinal cord injuries, which lead to limited orthostatic positioning in these patients.

**Objective:**

To investigate the cardiovascular and biomechanical parameters during 90 min of postural elevation equipment usage comparing spinal cord injury and healthy subjects.

**Methods:**

A device was used that allowed patients with spinal cord injuries to remain in an orthostatic posture for 90 min. During this period, the physiological parameters were measured every 15 min. Cardiovascular parameters (heart rate, oxygen saturation, blood pressure, and autonomic nervous system) and biomechanical parameters of the plantar pressure distribution were evaluated. For blood pressure, heart rate, oxygen saturation, and autonomic nervous system, a two-way analysis of variance was applied. The mixed-effect model was applied to plantar pressure. The significance level was set at *p* < 0.05 for all statistical analyses.

**Results:**

No differences were observed between the groups in systolic blood pressure (*F* = 0.07), diastolic blood pressure (*F* = 0.14), heart rate (*F* = 0.56), and oxygen saturation (*F* = 0.23) at any of the time intervals throughout the experiment (*p* > 0.05). No statistical difference was observed in the mean plantar pressure values between the groups (*p* = 0.35) during the period in which they remained in the orthostatic position.

**Conclusion:**

The present study showed the absence of differences between spinal cord injury patients and control participants using the orthostatic device in terms of cardiovascular and biomechanical parameters over 90 min.

## 1. Introduction

Spinal cord injury (SCI) can affect the social participation of individuals, as it leads to motor changes such as paralysis, musculoskeletal injuries, pain, and osteoporosis [1]. Individuals with SCI need medical care and rehabilitation, which involve access to wheelchair-friendly environments and appropriate homecare equipment, transport, employment, and financial support [1]. Individuals with limited mobility show limited participation in social and community activities and are associated with the severity of depressive symptoms [2–4].

Another factor that contributes to limiting the return of patients with SCI to social and work contexts is the presence of postural hypotension. SCI leads to peripheral and central cardiovascular adaptations such as increased peripheral vascular resistance, reduced capillarization, and decreased artery diameters, which cause static hypotension and limits a standing position [5]. Orthostatic hypotension affects patients with SCI and is defined as a decrease in systolic blood pressure (BP) >20 mmHg or diastolic BP > 10 mmHg within three minutes of becoming an upright posture [6]. Changes in autonomic function are often caused by spinal sympathetic control, influencing BP stability, including hypotension during rest and severe drops in BP during orthostatic positioning [7]. Considering these alterations, patients with SCI remain in the orthostatic position for a maximum of 15 minutes without alterations in BP or the presence of postural hypotension [8–10]. Changes in BP can also lead to lower limb involvement, such as edema, which is reported in approximately 45% of patients with SCI, resulting in limited joint mobility and range of motion and an increased risk of thromboembolism [11]. Therefore, it is important to measure the plantar pressure during long-term orthostatic positioning in patients with SCI. Orthopedic and functional orthoses and exoskeletons are often used to support and minimize physical limitations [11]. Additionally, they can be defined as external mechanical structures that are wearable and capable of providing support to the affected body segments of disabled people [12, 13]. This can increase the autonomy in movements, which can be positively explored in the job market, especially in the industrial field [11–13]. The use of exoskeletons for people with disabilities began in the 60 s. However, the equipment used in health and rehabilitation is limited owing to the complexity of use [12], difficulties in reproducing the prototypes, and limitations of clinical trials to validate their effectiveness for vulnerable populations [12, 13]. To the best of our knowledge, this is the first study to evaluate the physiological and biomechanical parameters of subjects with SCI in an orthostatic position over a long period. Therefore, more studies are needed to highlight the risks and benefits of using postural elevation equipment for an extended period to help include device use in the laboral context of SCI patients. This study aimed to investigate cardiovascular and biomechanical parameters during 90 min of use of postural elevation equipment in healthy and SCI subjects.

## 2. Methods

### 2.1. Description of the Research Subjects

This cross-sectional experimental study was approved by the Local Committee of Ethics in Research (number CAAE 07192819.6.0000.5038). Due to the nature of the injury condition, a priori sample calculation was not performed. Thus, the participants were intentionally selected for the study.

The following individuals were included in the control and SCI groups: individuals between 18 and 50 years, with a height between 1.55 and 1.75 meters, a maximum weight of 100 kg, of both sexes, healthy, without associated vascular pathologies (disorders of the coagulation, decompensated diabetes, etc.), and with stabilized BP. Those with any serious cognitive/psychological impairment that could interfere with test performance, such as panic syndrome, major depression or anxiety attacks, and any relevant speech impairment that could impede their communication during the tests.

Participants without motor changes were included in the control group. In the SCI group, subjects with medium and lower lesions due to trauma, accidents, gunshot, and others, without associated vascular pathologies (clotting disorders, decompensated diabetes, etc.), with stabilized BP.

### 2.2. Description of the Equipment

**Figure 1:**
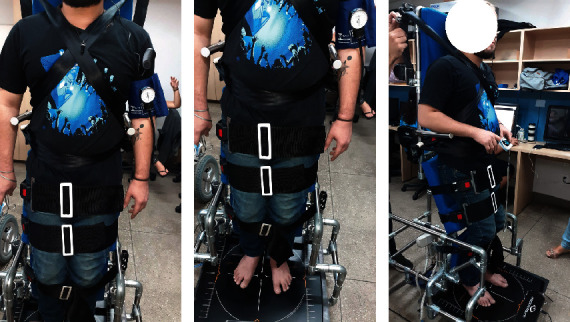
(a) Trunk bands; (b) leg bands; (c): side view—stand position.

The equipment (Figure 1(a)) was developed with AISI 409 stainless steel tubes, 28 mm in diameter and 0.7 mm thick joints, and articulated connections (Figure 1(b)). The participants were positioned in the orthostatic device and stabilized using adjustable straps on the shoulders, trunk, and hips. A wider Velcro band was added in the region of the legs (above the knee) and tibialis anterior. These bands allow greater stability of the lower limbs in the standing posture. Once positioned, the device was elevated, allowing the participant to remain standing. Similar to other wheelchairs, they can do this using a joystick. While elevated, users can still use the joystick to make fine personal adjustments to search for a more comfortable position. When the user is ready to leave the standing position, she presses the button and keeps it pressed until the device returns to its sitting position and can be used again as a regular motorized wheelchair. At the base of the equipment, a mechanism was installed that allowed ankle dorsiflexion movement to generate joint mobility during the orthostatic position (circulatory support) (Figure 1(c)).

### 2.3. Data Collection and Description

The participants were recruited for convenience, and a self-report sleep quality questionnaire and demographic characteristics were collected. The weight of each participant was then checked on a digital scale, and the first measurement of the autonomic nervous system activity in the sitting position was performed. Those to be evaluated were then positioned in the exoskeleton and fixed with belts specifically designed to guarantee user safety. After positioning each subject, they were placed in a condition of elevation with the exoskeleton (standing up position wearing the equipment) and maintained for 90 min. In this condition, the participants performed a battery of tests, which consisted of analysis of their heart rate, oxygen saturation, BP every 15 min, quantification of the activity of the autonomic nervous system, weight distribution on the equipment, and distribution of the plantar pressure on the baropodometry equipment every 30 min. Then, the users were removed from the elevated condition and returned to the sitting position, and the test was finished. Sleep quality data were collected to verify whether patients had any changes since vagal modulation is observed during sleep and sympathetic control is predominant during rapid eye movement (REM) sleep [14].

### 2.4. Cardiovascular Parameters

The participants' cardiovascular parameters (BP, heart rate, and oximetry) were inspected nine times at intervals of 15 min during and after the intervention. BP was assessed using a manual sphygmomanometer (BIC Brand™) that was properly calibrated and validated [13]. The cuff had a Velcro for fixation and a device that allowed the equipment to be inflated, which was positioned in the proximal region of the arm of the participants. The oximeter (BIC Brand™) was positioned on the finger, and oxyhemoglobin and deoxyhemoglobin were measured by red and infrared light [15]. The autonomic nervous system was evaluated using the InnerBalance(TM) at baseline, 30 min, 60 min, and 90 min of equipment use. The measurement of neurocardiac function reflects the dynamics of the autonomic nervous system (ANS), with measurements of the oscillations of the R–R intervals. EmWave(TM) software (Quantum Intech, Inc. Boulder Creek, CA, USA) was designed by the Institute of Heart Math. Data were processed using Kubios HRV(TM) version 2.1. The parameters analyzed were the sympathetic nervous system index (SNS) and parasympathetic nervous system index (PNS) were analyzed [16, 17].

### 2.5. Biomechanical Parameters

Before baropodometry, the subjects were weighed using a Renpho (TM) digital scale. Plantar pressure mean analysis was performed using a baropodometer (T-Plate, Medicapteurs (TM), France). Data of the plantar pressure mean they were collected for 50 s at four different moments during the period when the subjects were in the elevated position: one at Time 0, the second at 30 min, the third at 60 min, and the last at 90 min [18, 19].

### 2.6. Data Analysis

Data were analyzed using the statistical software GraphPad Prism 8.0. For the variables age, height, and body mass index, Student's *t*-test for Independent Samples was used. Data on weight and sleep quality showed a non-normal distribution, which is why they were evaluated using the Mann–Whitney *U* test. The groups were compared at the respective times (baseline, 15, 45, 60, 75, and 90 min), and the Shapiro-Wilk normality test was used. For BP, heart rate, oxygen saturation, and ANS, a two-way analysis of variance (ANOVA) was applied. The mixed-effect model was applied to plantar pressure. The significance level was set at *p* < 0.05 for all statistical analyses. Data are expressed as the mean (SD).

## 3. Results

### 3.1. Sample Characterization

The group samples had similar characteristics at baseline. No statistically significant differences were observed (Table 1).

### 3.2. Cardiovascular Parameters

No differences were observed between the groups in systolic blood pressure (*F* = 0.07), diastolic blood pressure (*F* = 0.14), heart rate (*F* = 0.56), and oxygen saturation (*F* = 0.23) at any of the time intervals throughout the experiment (*p* > 0.05) (Table 2). This could characterize the average clinical stability in the cardiovascular functions during the use of the exoskeletal postural elevation, with regard to the systolic and diastolic BP (with the mean below or until 120/80 mmHg), maximum heart rate (below 100 bpm), and oxygen saturation (above 95%).

### 3.3. Biomechanical Parameters

**Figure 2:**
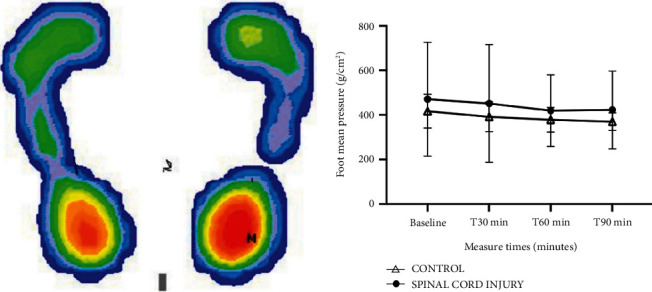
Postural analysis of the average plantar pressure of the evaluated individuals during the intervention period with the exoskeleton. (a) Image of the distribution of the average plantar pressure in the 50 s evaluation interval. (b) Average plantar pressure values every 30 minutes of wearing the exoskeleton in control and spinal cord groups.

Figure 2 shows the postural analysis of the average plantar pressure of the evaluated individuals. No statistical difference was observed in the mean plantar pressure values between the groups (*p* = 0.35) during the period in which they remained in the orthostatic position.  Table 3 shows the activity of the ANS based on heart rate variability, and no changes were observed between the control and paraplegic groups in the sympathetic and parasympathetic values throughout the study period (*p* > 0.05).

## 4. Discussion

This study aimed to evaluate the influence of the use of orthostatic equipment for 90 min on cardiovascular parameters, ANS, and plantar pressure in patients with SCI compared to controls. Our results showed the stability of cardiovascular parameters throughout using the exoskeleton for postural elevation. Additionally, the ANS responses were similar in both groups, and the plantar pressure mean always remained equal between the groups. In contrast, the time adaptation of this data over time demonstrated a nonspecific intragroup difference.

The cardiovascular parameters measured by heart rate, BP, and oxygen saturation in the blood demonstrated that none of the participants presented values that characterized the presence of dysfunction, such as BP [20]. This is an interesting fact, as it differs from a previous study [8] that demonstrated increased BP in paraplegic patients with incomplete SCI during wheelchair elevation for only 15 min [21, 22]. Orthostatic positions in patients with SCI are challenging because BP regulation is required due to orthostatic stress, which generates a displacement of fluids to the lower limbs and abdomen, thus reducing venous return to the heart [23, 24]. This expected response is called autonomically mediated baroreflex, which increases heart rate, contractility, and sympathetic vasoconstriction [21, 22].

The equipment developed and tested in this study had a circulatory support system. The base of the device, where the patient's feet were supported, was designed to move approximately 15° in ankle dorsiflexion, allowing joint mobility and consequently improving venous return and edema. The same system has already been tested in amputees who practiced physical activity for 90 min and showed favorable BP results [25]. Another important point to note is that the use of hip, trunk, and leg fixation straps may have led to greater comfort and a sense of security for the patient, which may have reduced the perception of factors such as anxiety and fear of falling off the floor of the equipment, contributing to greater stability of cardiovascular parameters.

Changes in the ANS can lead to changes in heart rate variability, as heart–brain interactions are essential for vital functions [26]. In this context, SCI can lead to autonomic disorders that increase the risk of developing cardiovascular changes and imbalance between the sympathetic and parasympathetic nervous systems [27]. The literature indicates that autonomic function can improve after exercise, but recommendations on workload monitoring and activity prescription for this population are still scarce [27]. Recent findings by our research group indicate that amputees who practice physical activity can also remain in the orthostatic posture using the same equipment proposed in this study, for 90 min and without changes in autonomic functions [25]. Thus, our data add to the literature the feasibility of using the device and the permanence of the patient with SCI in orthostatism for 90 min in the parameters in question.

The postural control system works such that the upright posture is maintained with the lowest possible energy expenditure, providing control, stability, and balance [28]. Thus, the measurement of plantar pressure was necessary to evaluate the distribution of load in the different plantar regions, as patients with SCI have limited joint mobility and reduced venous return, which can lead to the presence of edema and consequently an alteration in the distribution of pressure throughout the foot [29] during the period of use of the equipment. Patients with SCI have a higher risk of developing pressure ulcers caused by motor and sensory changes, skin changes, and prolonged periods of immobility [29], which were not observed in the feet of the evaluated patients. Thus, the data from this study demonstrate that standing for 90 min did not generate changes in the plantar pressure of patients with SCI compared with the control over the time of use.

### 4.1. Study Limitations and Strengths

The results in both groups concerning the stability of the cardiovascular parameters during device use for 90 min represent the main strength of this study. Using orthostatic equipment or wheelchair elevation devices could represent a new, safe, and effective technology to help individuals with paraplegia assume a standing position, thus allowing them more mobility [28]. On the other hand, our study has shown that the cardiovascular and postural effects of using orthostatic equipment during long-term continuous conditions, such as work journeys, have not yet been studied. Another limitation was the impossibility of using continuous BP and heart rate monitoring equipment, which prevented us from having more effective control over the variation of these parameters at all times.

### 4.2. Future Directions

This study demonstrates physiological stability for using the exoskeleton by healthy individuals and individuals with SCI for 90 min. Considering that exoskeletons allow overcoming environmental barriers, acting as part of a rehabilitation process [26], this study opens the possibility of verifying the neurophysiological aspects of the continuous long-term use of the device. In addition, it can boost new studies with more vulnerable populations, such as quadriplegics, hemiplegics, and patients with neurological syndromes, both at home, at work, and in a therapeutic context.

## 5. Conclusions

The present study showed the absence of difference between SCI patients and control participants using the orthostatic device in terms of cardiovascular and biomechanical parameters (heart rate, oxygen saturation, BP, and plantar pressure distribution) for 90 min, allowing, in clinical practice, the patient to remain in an orthostatic position for longer than reported so far in the literature.

## Figures and Tables

**Table 1:** Clinical and demographic characteristics.

	Control (*n* = 15)		Spinal cord injury (*n* = 15)		*p* value
Sex (male) (*n*)	7		11		
	Mean	(SD)	Mean	(SD)	
Age (years)	26.20	(7.25)	31.33	(8.42)	0.08
Weight (kg)	71.47	(15.5)	64.73	(14.35)	0.22
Height (m)	1.66	(0.10)	1.67	(0.08)	0.93
BMI (kg/cm^2^)	25.61	(3.59)	23.25	(3.87)	0.09
Sleep quality (0–10)	6.70	(2.58)	8.23	(1.16)	0.06

Abbreviations: kg: kilogram; m: meters; SD: standard deviation; BMI: Body Mass Index.

**Table 2:** Comparison of cardiovascular parameters over 90 min of maintaining the orthostatic position.

	Control (*n* = 15)	Spinal cord injury (*n* = 15)		
	Mean (SD)	Mean (SD)	*p* value	Mean difference (95% CI)
**Systolic arterial pressure (mmHg)**				
Time 0	118.0 (10.1)	117.3 (13.3)	>0.99	–0.667 (–13.26 to 11.93)
Time 15 minutes	116.7 (11.7)	114.8(15.4)	0.99	–1.867 (–16.47 to 12.74)
Time 30 minutes	114.7 (13.0)	115.3 (16.4)	>0.99	0.667 (–15.05 to 16.38)
Time 45 minutes	117.3 (13)	114.7 (19.2)	0.99	–2.667 (–20.32 to 14.98)
Time 60 minutes	116.7 (9.7)	114.7 (18.4)	0.99	–2.000 (–18.00 to 14.00)
Time 75 minutes	116.7 (12.3)	115.3 (17.6)	>0.99	–1.333 (–17.59 to 14.92)
Time 90 minutes	116.0 (11.8)	115.0 (17.8)	>0.99	–1.000 (–17.18 to 15.18)
**Diastolic arterial pressure (mmHg)**				
Time 0	76.7 (10.5)	79.3 (9.6)	0.98	2.667 (–7.95 to 13.29)
Time 15 minutes	78.0 (10.8)	79.3 (12.8)	>0.99	1.333 (–11.22 to 13.88)
Time 30 minutes	74.7 (9.1)	80.0 (13.1)	0.8	5.333 (–6.712 to 17.38)
Time 45 minutes	78.3 (9.6)	78.7 (14.6)	>0.99	–0.667 (–13.87 to 12.53)
Time 60 minutes	79.3 (8.0)	78.7 (15.5)	>0.99	–0.667 (–14.06 to 12.73)
Time 75 minutes	78.0 (9.4)	80.0 (16.0)	0.99	2.000 (–12.15 to 16.15)
Time 90 minutes	78.0 (10.1)	78.7 (15.0)	>0.99	0.667 (–13.05 to 14.38)
**Heart rate (bpm)**				
Time 0	86.6 (22.4)	91.7 (19.3)	0.99	5.067 (–17.10 to 27.24)
Time 15 minutes	85.9 (12.9)	94.8 (15.7)	0.52	8.933 (–6.349 to 24.22)
Time 30 minutes	85.9 (16.1)	86.9 (17.6)	>0.99	0.9333 (–16.92 to 18.78)
Time 45 minutes	87.9 (14.0)	91.6 (19.4)	0.99	3.667 (–14.31 to 21.65)
Time 60 minutes	81.7 (9.9)	89.7 (15.8)	0.55	8.000 (–6.149 to 22.15)
Time 75 minutes	86.6 (13.2)	87.8 (15.7)	>0.99	1.200 (–14.10 to 16.50)
Time 90 minutes	87.1 (14.7)	84.6 (15.0)	0.99	–2.533 (–18.16 to 13.09)
**Oxygen saturation (%)**				
Time 0	96.9 (1.6)	97.1 (1.5)	0.99	0.267 (–1.422 to 1.955)
Time 15 minutes	96.7 (2.7)	96.8 (1.7)	>0.99	0.067 (–2.309 to 2.442)
Time 30 minutes	96.7 (1.9)	97.3 (1.3)	0.93	0.600 (–1.159 to 2.359)
Time 45 minutes	96.9 (1.5)	96.9 (2.5)	>0.99	0.000 (–2.261 to 2.261)
Time 60 minutes	96. (2.8)	97.4 (1.6)	0.89	0.933 (–1.505 to 3.372)
Time 75 minutes	97.0 (1.6)	96.9 (1.7)	>0.99	–0.067 (–1.863 to 1.730)
Time 90 minutes	97.7 (1.0)	97.1 (1.8)	0.93	–0.533 (–2.096 to 1.029)

SD = standard deviation; 95% CI = 95% of CI.

**Table 3:** Functional analysis of the autonomic nervous system.

	Control (*N* = 15)		Spinal cord injury (*N* = 15)			
	Mean	(SD)	Mean	(SD)	*p* value	Mean difference (95% CI)
**Sympathetic**						
*T (0)*	0.479	(0.924)	1.072	(1.232)	0.4735	0.593 (–0.471 to 1.66)
*T (30)*	1.092	(0.745)	1.000	(0.954)	0.9972	–0.092 (–0.927 to 0.743)
*T (60)*	1.175	(0.761)	1.256	(1.128)	0.9989	0.081 (–0.862 to 1.025)
*T (90)*	1.053	(0.771)	1.053	(1.241)	>0.9999	0.001 (–1.017 to 1.018)
**Parasympathetic**						
*T (0)*	0.702	(1.822)	0.309	(1.834)	0.9624	–0.394 (–2.170 to 1.382)
*T (30)*	–0.030	(1.575)	0.576	(1.983)	0.8347	0.606 (–1.140 to 2.352)
*T (60)*	–0.901	(2.487)	0.930	(2.274)	0.1677	1.831 (–0.4863 to 4.148)
*T (90)*	–0.013	(1.315)	0.418	(1.731)	0.9079	0.431 (–1.070 to 1.932)

SD = standard deviation; CI = confidence interval.

## Data Availability

The data can be found in the Local Committee of Ethics in Research (number CAAE 07192819.6.0000.5038) and access to raw data can be shared with reasons and contact with authors.

## Supplementary Materials

Supplementary Materials
**Table 02 –**Comparison of cardiovascular parameters over 90 minutes of maintaining the orthostatic position.
