# Supplementary material for: The Use of Orthostatic Device for 90 Minutes Does Not Change Cardiovascular and Biomechanical Parameters of Patients with Spinal Cord Injury
Source: Appl Bionics Biomech. 2022 Sep 16;2022:3917566. doi: 10.1155/2022/3917566 (PMC9507789; doi:10.1155/2022/3917566)
Supplement: Supplementary Materials — Table 02 –Comparison of cardiovascular parameters over 90 minutes of maintaining the orthostatic position. [file 3917566.f1.docx]

| **Table 02 –** Comparison of cardiovascular parameters over 90 minutes of maintaining the orthostatic position. | | | | | |
| --- | --- | --- | --- | --- | --- |
|  | Control  **(n=15)** | Spinal Cord Injury  **(n=15)** |  |  | |
|  | Mean (SD) | Mean (SD) | p value | Mean difference (95% CI) | |
| **Systolic Arterial Pressure (mmHg)** | | | | | |
| *Time 0* | 118.0 (10.1) | 117.3 (13.3) | >0.99 | -0.667  (-13.26 to 11.93) |  |
| *Time 15 minutes* | 116.7 (11.7) | 114.8(15.4) | 0.99 | -1.867  (-16.47 to 12.74) |  |
| *Time 30 minutes* | 114.7 (13.0) | 115.3 (16.4) | >0.99 | 0.667  (-15.05 to 16.38) |  |
| *Time 45 minutes* | 117.3 (13) | 114.7 (19.2) | 0.99 | -2.667  (-20.32 to 14.98) |  |
| *Time 60 minutes* | 116.7 (9.7) | 114.7 (18.4) | 0.99 | -2.000  (-18.00 to 14.00) |  |
| *Time 75 minutes* | 116.7 (12.3) | 115.3 (17.6) | >0.99 | -1.333  (-17.59 to 14.92) |  |
| *Time 90 minutes* | 116.0 (11.8) | 115.0 (17.8) | >0.99 | -1.000  (-17.18 to 15.18) |  |
| **Diastolic Arterial Pressure (mmHg)** | | | | | |
| *Time 0* | 76.7 (10.5) | 79.3 (9.6) | 0.98 | 2.667  (-7.95 to 13.29) | |
| *Time 15 minutes* | 78.0 (10.8) | 79.3 (12.8) | >0.99 | 1.333  (-11.22 to 13.88) | |
| *Time 30 minutes* | 74.7 (9.1) | 80.0 (13.1) | 0.8 | 5.333  (-6.712 to 17.38) | |
| *Time 45 minutes* | 78.3 (9.6) | 78.7 (14.6) | >0.99 | -0.667  (-13.87 to 12.53) | |
| *Time 60 minutes* | 79.3 (8.0) | 78.7 (15.5) | >0.99 | -0.667  (-14.06 to 12.73) | |
| *Time 75 minutes* | 78.0 (9.4) | 80.0 (16.0) | 0.99 | 2.000  (-12.15 to 16.15) | |
| *Time 90 minutes* | 78.0 (10.1) | 78.7 (15.0) | >0.99 | 0.667  (-13.05 to 14.38) | |
| **Heart Rate (bpm)** | | | | | |
| *Time 0* | 86.6 (22.4) | 91.7 (19.3) | 0.99 | 5.067  (-17.10 to 27.24) | |
| *Time 15 minutes* | 85.9 (12.9) | 94.8 (15.7) | 0.52 | 8.933  (-6.349 to 24.22) | |
| *Time 30 minutes* | 85.9 (16.1) | 86.9 (17.6) | >0.99 | 0.9333  (-16.92 to 18.78) | |
| *Time 45 minutes* | 87.9 (14.0) | 91.6 (19.4) | 0.99 | 3.667  (-14.31 to 21.65) | |
| *Time 60 minutes* | 81.7 (9.9) | 89.7 (15.8) | 0.55 | 8.000  (-6.149 to 22.15) | |
| *Time 75 minutes* | 86.6 (13.2) | 87.8 (15.7) | >0.99 | 1.200  (-14.10 to 16.50) | |
| *Time 90 minutes* | 87.1 (14.7) | 84.6 (15.0) | 0.99 | -2.533  (-18.16 to 13.09) | |
| **Oxygen Saturation (%)** | | | | | |
| *Time 0* | 96.9 (1.6) | 97.1 (1.5) | 0.99 | 0.267  (-1.422 to 1.955) | |
| *Time 15 minutes* | 96.7 (2.7) | 96.8 (1.7) | >0.99 | 0.067  (-2.309 to 2.442) | |
| *Time 30 minutes* | 96.7 (1.9) | 97.3 (1.3) | 0.93 | 0.600  (-1.159 to 2.359) | |
| *Time 45 minutes* | 96.9 (1.5) | 96.9 (2.5) | >0.99 | 0.000  (-2.261 to 2.261) | |
| *Time 60 minutes* | 96. (2.8) | 97.4 (1.6) | 0.89 | 0.933  (-1.505 to 3.372) | |
| *Time 75 minutes* | 97.0 (1.6) | 96.9 (1.7) | >0.99 | -0.067  (-1.863 to 1.730) | |
| *Time 90 minutes* | 97.7 (1.0) | 97.1 (1.8) | 0.93 | -0.533  (-2.096 to 1.029) | |
| SD= Standard deviation; 95% CI = 95% of CI | | | | | |
